# Partner satisfaction or changes in partner satisfaction: A retrospective observational study

**DOI:** 10.1097/MD.0000000000046844

**Published:** 2025-12-26

**Authors:** Elif Ucar, Ozan Dogan, Pinar Kadirogullari, Erhan Huseyin Comert, Murat Yassa

**Affiliations:** a Department of Midwifery, Faculty of Health Sciences, Istanbul Esenyurt University, Istanbul, Turkey; b Department of Obstetrics and Gynecology, Women’s Health Clinic, İstanbul Nişantasi University, Pelvic Floor and Cosmetic Gynecology Association (PET-KOZ), Istanbul, Turkey; c Department of Obstetrics and Gynecology, Acibadem University Atakent Hospital, Pelvic Floor and Cosmetic Gynecology Association (PET-KOZ), Istanbul, Turkey; d Women’s Health Clinic, Pelvic Floor and Cosmetic Gynecology Association (PET-KOZ), Istanbul, Turkey; e Department of Obstetrics and Gynecology, Acibadem University Kartal Hospital, Pelvic Floor and Cosmetic Gynecology Association (PET-KOZ), Istanbul, Turkey.

**Keywords:** aesthetic, Golombok–Rust scale, MSHQ, partner, satisfaction, sexuality, vaginoplasty

## Abstract

In recent years, due to the increasing demand, genital aesthetic surgeries, particularly vaginoplasty, have become commonly preferred by women. However, data on the effects of this operation on male sexuality are limited. In recent years, societal awareness of sexual compatibility between partners and the surgical interventions that can be performed on the genital area to improve this compatibility has increased. This study aimed to demonstrate changes in partner satisfaction before and after vaginoplasty performed for aesthetic or medical reasons. This study was planned retrospectively. The sampling method used in the study was purposive sampling. Among 200 patients who underwent vaginoplasty, 47 were selected based on specific inclusion and exclusion criteria such as penis size perception, sexual activity, and the absence of erectile dysfunction. Patient information was screened from our clinic’s database, and demographic data were recorded. Only the partners of women who underwent vaginoplasty were included in the study. Among these partners, only those with an average or above-average penis size were included. The partners of patients who underwent vaginoplasty were interviewed to assess their sexual functions using the Male Sexual Health Questionnaire-Ejaculation Disorder (MSHQ-EjD), the Golombok–Rust sexual satisfaction scale, and the new sexual satisfaction scale (NSSS). These assessments were conducted in the preoperative and postoperative periods, and the data were recorded. In this study, it was observed that there were no significant differences in MSHQ ejaculation function and ejaculation difficulty levels between the preoperative and postoperative periods. The Golombok–Rust sexual satisfaction scale assessed the quality of the relationship in 8 subgroups, finding that levels of frequency, communication, touching, satisfaction, impotence, and quality of sexual intercourse were significantly higher postoperatively, while scores for avoidance and premature ejaculation were significantly lower. This study found that sexual satisfaction increased in the partners of women who underwent vaginoplasty for aesthetic or medical reasons.

## 1. Introduction

Sexual health and compatibility are important components of a healthy life and a fulfilling relationship with a partner. However, women’s discomfort with their genital appearance can cause anxiety and reticence during sexual intercourse.^[1]^ Both body image and sexuality are shaped not only by individuals but also by interpersonal and social experiences. Negative body image is thought to reduce sexual desire and arousal and is therefore associated with lower sexual satisfaction.^[2]^ It has been suggested that the main reason women seek gynecological cosmetic surgery is the belief that the surgery can improve their body image, sexual satisfaction, and marital relationships.^[3]^

A woman’s sexual function is influenced by factors such as previous vaginal deliveries, aging, genetic factors, relationships with partners, and other psychosocial factors. These factors can lead to various disorders ranging from sexual dissatisfaction to actual deviations.^[4]^ Vaginal delivery after vaginal trauma can cause stretching of vaginal tissues, separation of pelvic floor muscles, and vaginal laxity in women. This results in a wide and open perineum, reducing the sense of friction and sexual satisfaction.^[5]^ Women also frequently complain about the opening of the vaginal vestibule, which causes the vaginal mucosa to protrude.^[6]^ This opening leads to functional concerns, such as increased vaginal secretions due to exposed mucosa, changes in reaching orgasm, and vaginal air entrapment, which causes embarrassing noises during intercourse.^[7]^ Vaginal relaxation and expansion, especially common among women who have had vaginal deliveries, are thought to reduce pleasure during intercourse for partners and cause anxiety, decreased self-confidence, and depression in women.^[8,9]^ This condition may result in reduced sexual sensitivity and avoidance of sexuality in most women, and problems such as sexual reluctance and impotence in their partners.^[10]^ Due to physical discomfort and cosmetic concerns, many women perceive feelings of vaginal and perineal laxity as detrimental to sexual pleasure and ease of orgasm and seek vaginoplasty.^[1]^ While operations on the vagina were historically performed to treat congenital genital syndromes such as ambiguous genitalia, adrenogenital syndrome, and vaginal agenesis,^[11]^ increasing demand and awareness have transformed the procedure into 1 that reconstructs the vaginal lumen with appropriate axis, length, and width to ensure penis-vagina compatibility, enhancing sexual satisfaction, pleasure, and fulfillment.^[12]^

Information regarding the sexual satisfaction of partners of women seeking gynecological cosmetic surgery is insufficient. Therefore, this study aimed to compare not only the body image, sexual function, and satisfaction of women before and after vaginoplasty but also the sexual satisfaction of their partners.

## 2. Materials and methods

This retrospective study was conducted at a private clinic in Istanbul between January 2020 and January 2023. The study protocol was approved by the ethics committee of İstanbul Esenyurt University (March 16, 2023 – 2023/03-4). The records of 200 patients who underwent vaginoplasty were reviewed. Based on 3 years of data, 47 partners of patients were included in the study after face-to-face interviews. The sampling method used in the study was purposive sampling. Among 200 patients who underwent vaginoplasty, 47 were selected based on specific inclusion and exclusion criteria such as penis size perception, sexual activity, and the absence of erectile dysfunction. This approach ensured the inclusion of participants who could meaningfully evaluate the sexual outcomes related to the procedure.

Partners and patients were shown penis models prepared in accordance with the average penis sizes in Turkey and were asked to compare these with their own penises. A study by Aslan et al found that the average penis length in Turkey is 13.7 ± 1.6 cm.^[13]^ Patients who reported an average or above-average penis size were included in the study. Partners of patients who underwent surgeries other than vaginoplasty, were sexually active without a partner, were no longer sexually active due to age, or had a history of vaginismus or psychological treatment were excluded. Those who provided inconsistent answers between the penis model and their own penis size were also excluded. Partners of patients with below-average penis size, micropenis, or erectile dysfunction were not included.

The partners of the included women were interviewed separately in a private room. They completed the Male Sexual Health Questionnaire-Ejaculation Disorder (MSHQ-EjD), the Golombok–Rust sexual satisfaction scale, and the new sexual satisfaction scale (NSSS).^[14–16]^ The MSHQ-EjD is a 4-item questionnaire assessing male ejaculatory functions, with the first 3 questions evaluating ejaculatory function and the fourth assessing ejaculatory difficulty. The total score ranges from 1 to 15 for ejaculatory function, and 0 to 5 for ejaculatory difficulty, with higher scores indicating better sexual health. The Golombok–Rust sexual satisfaction scale consists of 28 questions that evaluate various sexual problems, including relationship frequency, communication, avoidance, touch, satisfaction, impotence, premature ejaculation, and the quality of sexual intercourse. Scores of 4 and above in the frequency and communication categories suggest problems in those areas, while scores of 9 and above in the other categories indicate issues. The NSSS consists of 20 questions assessing sexual satisfaction, with possible scores ranging from 10 to 100, with higher scores indicating greater satisfaction.

### 2.1. Surgical procedure

The same surgical procedure was performed on all patients included in the study by the same surgeon. The procedure involved making a posterior vaginal wall incision from the vaginal introitus to the posterior sacrouterine ligament level of the cervix, followed by dissection of the rectovaginal fascia. The defective rectovaginal fascia was repaired and fixed to the posterior cervix, completing the reconstruction of the cervical ring. Excessively dilated vaginal mucosa was excised, and the incision was continuously closed with 2.0 vicryl sutures.

### 2.2. Statistical analysis

Continuous variables were expressed as mean ± standard deviation and/or median (min–max), while categorical data were expressed as numbers and percentages. The normality of continuous variables was assessed using the Kolmogorov–Smirnov Goodness of Fit test. Preoperative and postoperative comparisons were conducted using paired samples *t*-test for normally distributed data and the Wilcoxon signed rank test for non-normally distributed data. The McNemar test was used to compare categorical variables. Analyses were performed using IBM SPSS version 26.0 (IBM Corporation, Armonk), with statistical significance set at *P* < .05.

#### 2.2.1. Power analysis

In the study of 47 participants, the minimum required sample size was calculated using GPower based on the means and standard deviations of preoperative and postoperative variables presented in Table 5. The Cohen *d* effect size was found to be 0.99. Entering a two-tailed test, an error margin of 0.05, and a power of 0.80 into GPower, the minimum sample size required was determined to be 11 participants. Given that 47 participants were included in the survey study, the sample size was deemed sufficient based on the power analysis.

## 3. Results

The demographic data, gynecological, and obstetric histories of the patients examined in the study are shown in Table 1. The average age of first sexual activity was 23.57 ± 4.86 years, and the average duration of marriage was 15.09 ± 8.11 years. It was found that 93.6% of patients had only 1 partner, 19.1% were menopausal, 93.6% lived with their spouse/partner, 68.1% were married by agreement, and 55.3% had an extended family (Table 2). The demographic data of the patients’ partners are presented in Table 3.

**Table 1:** Some descriptive data on patients.

	Patient group (n = 47)
Age (yr) (Mean ± SD)	39.34 ± 6.49
BMI (kg/m^2^) (Mean ± SD)	25.18 ± 3.87
Education level (n, %)	
Primary	11 (23.4)
High school	19 (40.4)
University	15 (31.9)
Master	2 (4.3)
Socioeconomic level (n, %)	
Income less than expenses	2 (4.3)
Income equal to expenses	35 (74.5)
Income more than expenses	10 (21.5)
Clothing (n, %)	
Without hijab	33 (70.2)
Hijab	13 (27.7)
Sheet	1 (2.1)
Smoking (n, %)	
Not using	30 (63.8)
Using	17 (36.2)
Systemic disease (n, %)	
None	41 (87.2)
Yes	6 (12.8)
Postoperative time (mo) (Mean ± SD)	12.46 ± 6.71
Parity (Median [min–max])	2 (0–7)
Number of children born with NVD (Median [min–max])	1 (0–6)
Number of children born with CS (Median [min–max]) (Mean ± SD)	0 (0–2) 0.45 ± 0.71
Abort number (Median [min–max]) (Mean ± SD)	0 (0–2) 0.28 ± 0.54
Number of living children (Median [min–max])	2 (0–7)
Previous gynecological operation (n, %)	
No	39 (83.0)
Yes	8 (17.0)
Non-cesarean section gynecological operation (n, %)	
None	45 (95.7)
Yes	2 (4.3)

BMI = body mass index, CS = C-section, NVD = normal vaginal delivery.

**Table 2:** Some characteristics of the family life of the patients.

	Patient group (n = 47)
Age at first sexual activity (yr) (Mean ± SD)	23.57 ± 4.86
Marriage duration (yr) (Mean ± SD)	15.09 ± 8.11
Number of partners (n, %)	
1	44 (93.6)
2	1 (2.1)
3	2 (4.3)
Menopause status (n, %)	
Menopause	9 (19.1)
Premenopausal	38 (80.9)
Marriage status (n, %)	
Single/widowed/divorced	3 (6.4)
Married/living together	44 (93.6)
How did she get married (n, %)	
Not married	2 (4.3)
Agreed	32 (68.1)
Arranged	13 (27.7)
Family structure (n, %)	
Not married	2 (4.3)
Core family	19 (40.4)
Extended family	26 (55.3)

**Table 3:** Some characteristics of the spouses of the patients.

	Patient group (n = 47)
Partner’s age (yr) (Mean ± SD)	43.02 ± 6.36
BMI (kg/m^2^) (Mean ± SD)	26.47 ± 3.67
Education level	
Primary school	8 (17.0)
High school	16 (34.0)
University	23 (48.9)
Systemic disease (n, %)	
None	46 (97.9)
Yes	1 (2.1)
Past operation (n, %)	
None	46 (97.9)
Yes	1 (2.1)

BMI = body mass index.

No significant differences were found in MSHQ ejaculatory function and ejaculatory difficulty levels between the preoperative and postoperative periods (*P* = .172 and *P* = .317, respectively) (Table 4).

**Table 4:** Comparison of preoperative and postoperative MSHQ-EjD ejaculation function and ejaculation difficulty levels.

	Preoperative (Median [min–max])	Postoperative (Median [min–max])	*P*
MSHQ ejaculation function	14 (3–15)	14 (3–15)	.172*
MSHQ difficulty ejaculation	5 (1–5)	5 (1–5)	.317*

MSHQ-EjD = Male Sexual Health Questionnaire-Ejaculation Disorder.

* Wilcoxon signed ranks test.

When comparing preoperative and postoperative Golombok levels, postoperative frequency, communication, touching, satisfaction, impotence, and quality of sexual intercourse levels were found to be significantly higher than preoperative values (*P* < .001 for all). In contrast, avoidance and premature ejaculation scores significantly decreased (*P* = .004 and *P* = .002, respectively) (Table 5).

**Table 5:** Comparison of preoperative and postoperative Golombok–Rust scores.

	Preoperative (Median [min–max])	Postoperative (Median [min–max])	*P*
Golombok – frequency	2 (1–6)	0 (0–5)	<.001*
Golombok – communication	3 (1–6)	1 (1–4)	<.001*
Golombok – avoidance	2 (1–6)	3 (0–5)	.004*
Golombok – touch	3 (0–7)	0 (0–6)	<.001*
Golombok – satisfaction	5 (3–10)	1 (1–9)	<.001*
Golombok – impotence	6 (4–7)	3 (2–5)	<.001*
Golombok – premature ejaculation	3 (2–7)	5 (1–6)	.004*
Golombok – quality of sexual intercourse	4 (1–6)	2 (2–7)	<.001*

* Wilcoxon signed ranks test.

Postoperative NSSS scores (85.89 ± 14.57) were found to be significantly higher compared to preoperative scores (73.11 ± 10.81) (*P* < .0001) (Table 6).

**Table 6:** Comparison of preoperative and postoperative NSSS scores.

	Preoperative (Mean ± SD)	Postoperative (Mean ± SD)	*P*
NSSS total	73,11 ± 10,81	85,89 ± 14,57	<.001*

NSSS = new sexual satisfaction scale.

* Paired samples *t*–test.

## 4. Discussion

The most common indication for vaginoplasty is vaginal laxity due to normal vaginal delivery and the natural aging process, which often leads to sexual dysfunction. While vaginoplasty, performed to improve sexual function, treat vaginal laxity, and address urinary incontinence, is becoming increasingly popular, scientific evidence remains insufficient. This study demonstrates that correcting vaginal relaxation and widening can enhance partner sexual satisfaction, potentially increasing couples’ overall sexual satisfaction and fulfillment. The literature indicates that dissatisfaction with partners and negative comments from partners are significant factors motivating aesthetic procedures for the female genital system.^[17,18]^ Some studies even highlight partner opinion as the most critical factor.^[19]^

Mutual pleasure is an essential component of sexual satisfaction, which arises from positive sexual experiences rather than the absence of conflict or dysfunction.^[20]^ Furthermore, sexual satisfaction is considered a crucial component of sexual health, a sexual right, and a result of sexual well-being.^[21]^ Studies have shown that a satisfying and safe sexual relationship is one of the most impactful dimensions of marital satisfaction. Thus, improving sexual satisfaction can enhance marital satisfaction.^[22–24]^ Therefore, we suggest that vaginoplasty may contribute to more stable marital relationships, emphasizing the importance of partner satisfaction. Studies have shown that when performed by experienced surgeons, gynecological aesthetic procedures positively impact women’s body image, aesthetic appearance, and functional improvement, reflecting positively on their lives.^[25]^ In our study, satisfaction of the partners of 47 women who underwent vaginoplasty was assessed through surveys, revealing significantly higher postoperative levels of sexual frequency, communication, touching, satisfaction, impotence, and quality of sexual intercourse, along with significantly lower avoidance and premature ejaculation scores.

Iglesia states that the concept of the “perfect vagina” is a significant aspect of female beauty.^[26]^ She argues that general satisfaction with one’s body leads women to be more open to new sexual experiences and more sexually willing. Furthermore, she claims that positive body image increases the frequency of sex and orgasm, making women feel more comfortable giving pleasure to their partners.^[27]^ When women seek gynecological cosmetic surgery, body image is often another source of concern. Body image-related parameters, such as excess weight, physical condition, sexual attractiveness, and thoughts about the body during sexual intercourse, predict sexual satisfaction. These findings suggest that women with low sexual satisfaction may benefit from treatments targeting specific aspects of body image.^[28]^ A study in the Netherlands found that interventions focused on developing and maintaining a positive body image could help establish a more satisfying sex life and higher relationship quality.^[3]^ The impact of various types of aesthetic surgery on body image is linked not only to physical appearance but also to mental well-being.^[29]^

It has been shown that high levels of genital and sexual dissatisfaction before female genital cosmetic surgeries significantly decrease postoperatively, indicating that dissatisfaction with a perceived defect is a motivator for surgery.^[1]^ About 40% of women experience psychological distress due to sexual dysfunction, yet only 14% of women consult a doctor about sex during their lifetime.^[30]^ Today, the rapid increase in access to information, medical access, and media exposure allows for earlier detection of sexual dysfunctions, leading to higher demand for cosmetic procedures such as vaginoplasty. In India, demand for aesthetic vaginal procedures rose from 3.9% in 2012 to 28.92% in 2015.^[31]^ In our study, 200 patients visited our clinic solely for vaginoplasty within 3 years, reflecting increasing demand and awareness.

Austin et al^[32]^ , in a study examining patients undergoing vaginoplasty, found the average patient age to be 41. In our study, the average age of patients was 39.34 ± 6.49 years, which aligns with the literature. Austin et al reported that the majority (86.7%) of patients undergoing vaginoplasty were multiparous, while the remaining 13.3% were primiparous.^[7]^ In our study, 68.1% (32 patients) of participants were multiparous, while 31.9% (15 patients) were primiparous, similar to previously reported rates. Previous studies have shown that most women seeking genital aesthetic surgery are housewives with primary education or have a bachelor’s degree, indicating significantly lower education levels.^[33]^ Additionally, Rowen et al found that older women with high school or higher education levels were less likely to report genital dissatisfaction.^[34]^ In our study, 76.6% (36 patients) had a high school or higher education level.

When considering functionality and beauty together, it is believed that the positive changes in the female body will also positively affect partners. Goodman et al found that most women who underwent genital cosmetic surgery were satisfied with their genital area, experienced positive changes in their sexuality postsurgery, and reported increased sexual activity.^[35]^ Eftekhar et al demonstrated increased sexual satisfaction among couples after genital aesthetic surgery, showing that male partners might also benefit from improvements in female sexual function.^[33]^ Pascoal et al emphasized that sexual satisfaction arises from mutual pleasure.^[20]^ Similarly, a multicenter study found that male partners’ satisfaction was notably high after genital aesthetic surgeries.^[35]^ In our study, although there were no significant differences in ejaculation function and difficulty levels in partners, the quality and nature of sexual intercourse were significantly higher in the postoperative period, with significantly lower avoidance and premature ejaculation scores.

In a multicenter cross-sectional study, 47 women underwent vaginoplasty/perineoplasty, showing high satisfaction rates in enhancing sexual function for both women and their partners.^[35]^ Most studies on patient satisfaction and sexual function after vaginal aesthetic and functional plastic procedures report positive outcomes, with overall patient satisfaction between 90% and 95% and sexual satisfaction above 80% to 85%.^[36]^ In a study conducted by Pardo et al, 94% of 53 women who complained of vaginal laxity and underwent vaginal tightening surgery reported experiencing a tighter vagina and higher orgasm rates 6 months later.^[37]^ Our results were similar to those of Pardo et al, showing high satisfaction rates for both patients and their partners. In a case-control study by Davai et al, marital satisfaction was higher in the surgery candidate and operated groups compared to the control group, indicating the impact of cosmetic surgery on marital satisfaction.^[38]^ These findings are consistent with the results of a study by Tavassoli and Modiri on the effects of cosmetic surgery on marital satisfaction. Women reported that the beauty provided by aesthetic surgery contributed to greater self-confidence, more success in marital life, higher social status, and prestige, and was a factor in achieving power within the family.^[39]^ This suggests that the increased partner satisfaction in sexual life post-vaginoplasty improves couples’ mutual harmony and positively influences the management of the marital process.

These surgeries enhance women’s self-confidence, positively affect their view of sexuality and sexual desire, and lead to a more active role in their sexual lives, which also positively impacts their partners. Consequently, the pleasure derived from sexuality as a couple increases. In this study, it was shown that using MSHQ, Golombok–Rust scale, and NSSS questionnaires, there was a statistically significant increase in partners’ postoperative sexual functions and relationship satisfaction. Elçi et al found that although not openly expressed between couples, late ejaculation and decreased sexual frequency led to a reduction in sexual pleasure.^[40]^ In this study, according to the MSHQ questionnaire, no significant difference was detected in male ejaculation function during sexual intercourse in the preoperative and postoperative periods. However, a statistically significant reduction in avoidance and premature ejaculation categories was observed postoperatively in the Golombok–Rust scale, suggesting that the frequency, quality, and satisfaction of the relationship are more important in ejaculation functions. This suggests that vaginal relaxation affects sexual pleasure but is not related to ejaculation function. Achieving sexual harmony increases couples’ communication, harmony, relationship quality, diversity, and frequency.^[41]^ In this study, the Golombok–Rust scale showed that sexuality, communication, harmony, relationship quality, diversity, and frequency were significantly higher in the postoperative period for partners.

Recent studies have emphasized the role of HPV and sexually transmitted infections in altering women’s perceptions of their genital appearance and sexual self-esteem. HPV-related anxiety, stigma, and physical changes (e.g., after wart treatment or conization procedures) may lead to increased concern about genital aesthetics. Consequently, the demand for genital cosmetic procedures such as vaginoplasty may be influenced not only by functional or sexual motives but also by psychological and social factors.^[42–44]^

## 5. Limitations and prospects

The strengths of the study include the application of the same surgical procedure by the same surgeon with specific experience in vaginoplasty surgery for all patients. This minimized the possibility of complications being attributed to different surgeons and surgical procedures, ensuring an objective evaluation of the success and complications of the surgical procedure. Our study is a pre-post study without a control group, limiting the establishment of a causal relationship. Additionally, psychological variables such as stress, depression, or anxiety, which could affect sexual function and satisfaction, were not assessed, representing another limitation. Nevertheless, the limited research on the impact of vaginoplasty on male partner satisfaction postoperatively highlights the significant contribution of this study to the literature.

Although the duration of sexual intercourse may be an important factor in overall satisfaction, it was not evaluated in this study and should be addressed in future prospective studies with appropriate tools.

## 6. Conclusion

An increase in sexual pleasure and satisfaction was observed among partners of patients who underwent vaginoplasty. However, no significant differences were found in ejaculatory functions between the preoperative and postoperative periods. It was demonstrated that vaginal relaxation and laxity negatively affect sexual pleasure and satisfaction in partners, and that these issues can be addressed through vaginoplasty, which has no impact on ejaculatory functions. The improvement in couples’ sexual satisfaction following vaginoplasty surgery indicates that enhancing sexual function in women can also benefit their male partners.

## Author contributions

**Conceptualization:** Elif Ucar, Murat Yassa.

**Data curation:** Elif Ucar, Ozan Dogan, Murat Yassa.

**Formal analysis:** Elif Ucar, Ozan Dogan, Erhan Huseyin Comert, Murat Yassa.

**Project administration:** Ozan Dogan.

**Supervision:** Pinar Kadirogullari.

**Visualization:** Erhan Huseyin Comert.

**Writing – original draft:** Elif Ucar.

**Writing – review & editing:** Pinar Kadirogullari, Murat Yassa.
